# Dry- down probe free qPCR for detection of KFD in resource limited settings

**DOI:** 10.1371/journal.pone.0284559

**Published:** 2023-05-10

**Authors:** Pooja Yadav, Shashi Sharma, Paban Kumar Dash, Suman Dhankher, Sandhya V. K., S. K. Kiran

**Affiliations:** 1 Virology Division, Defence Research & Development Establishment, Gwalior, India; 2 Virus Diagnostic Laboratory, Shivamogga, Karnataka, India; Huadong Research Institute for Medicine and Biotechniques, CHINA

## Abstract

Kyasanur Forest Disease is a tick-borne flavivirus is endemic in the Southern India. The recent expansion and resurgence of sporadic outbreaks in southern parts of country is the most important concern. Although only formalin inactivated vaccine is available for treatment with limited efficacy the early detection and timely identification is a only way to prevent spread of cases. If the disease can be identified prior to infection in humans like in forest areas from ticks and vectors the disease spread supposed to be managed quickly. Here we have standardized a single tube ready to use dry-down probe free real time RT-PCR targeted against virus envelope gene for detection of KFDV infection. The assay was standardized in liquid format first, later it was converted into dry-down format with addition of stabilizers with a similar sensitivity and specificity (10RNA Copies/rxn). The sensitivity was comparable to the most widely used and accepted diagnostic platform i.e. TaqMan qRT-PCR. However as the reported assay here omit the need of probes makes it cost effective and dry-down reagents makes more stability to the developed assay in this study if compare to TaqMan qPCR. The assay was evaluated with KFD positive samples and healthy sample panel which revealed high concordance with TaqMan qRT-PCR. Stability was unaffected by temperature fluctuations during transportation even in cold chain free conditions, thus reduce the maintenance of strict cold storage. These findings demonstrated that the reported assay is convenient with 100% sensitivity and specificity to TaqMan qPCR. Thus this assay has the potential usefulness for diagnosis KFDV for routine surveillance in resource limited laboratory settings omitting the use costly and heat sensitive TaqMan qRT-PCR reagents without compromising the sensitivity and specificity of the diagnosis assay.

## Introduction

Kyasanur Forest Disease (KFD) is an emerging tick-borne flavivirus haemorrhagic fever infection of immense public health concern in India especially southern India states affected Karnataka, Kerala, Tamil Nadu, Goa and Maharashtra [1, 2]. It was first identified in a sick monkey from Shivamogga district of Karnataka in 1957. In the current scenario, ecobiological changes caused emergence of KFD outside the endemic zone [3–5]. KFD is caused by Kyasanur Forest Disease Virus (KFDV). It is a single stranded positive sense enveloped RNA virus. It is a member of *Flavivirus* genus of family *Flaviviridae* and is principally transmitted to humans and animals by tick vector *Haemaphysalis spinigera*. KFDV is antigenically related to other tick borne flaviviruses and resembling Russian-Spring–Summer Complex of viruses. KFDV produces a febrile illness in humans that is often characterised by sudden onset of fever, chills, frontal headache, severe prostration, conjunctivitis, bleeding from nose, mouth, and gastrointestinal tract. The incubation last for ~3–8 days [6].

Currently, the diagnosis of KFD infection is accomplished by real time RT-PCR assay, nested RT- PCR assay, anti-KFD IgM and anti-KFD IgG ELISAs in a well established diagnostic laboratory [7]. But all these formats have their own limitations and sensitivity issues. The serological assay has limitations in terms of sensitivity whereas TaqMan based qPCR requires trained manpower, costly probes as well as high end equipments like real time PCR. None of them is available at a commercial platform and also lack onsite detection capabilities. It is difficult to train manpower for real time RT-PCR assay and reagents are very expensive and degrade very fast, in addition to this, sophisticated instruments are also required. This also obviates multiple pipetting steps that often lead to error-prone results. Thus the usefulness of the developed assays over real time PCR in remote areas, fast turnaround time, and subsequent quick patient management.

The TaqMan Real time RT-PCR is currently used for the KFDV detection in acute phase. However due to lack of ready to use PCR tool, trained manpower, molecular diagnosis is often delayed, because the samples must be sent to reference laboratories, which are frequently located far. There may also be significant difficulty obtaining and storing expensive reagents.

Therefore, the developed assay in this study has advantages over the other available detection platforms against KFD. The assay in dry-down format can easily adapted with a portable and define real time PCR in field settings. The ready to use viral RNA extraction methods quickly integrate with the downstream dry-down probe free PCR. Thus, the dry down probe free real time PCR offers a alternate detection method in KFDV diagnosis in resource limited settings.

Keeping in the view above facts, current study was formulated to check applicability of ready-to-use, dry-down qRT-PCR reagents for rapid diagnosis of KFD infections. This dry- down formulations provides stability to reagents against temperature fluctuation and storage conditions which is very critical for storage of qPCR reagents in molecular diagnosis.

## Materials and methods

### Clinical samples and ethical statement

The serum samples from patients (n = 50 with KFD infection during outbreaks in different parts of southern India from 2017–2019 were collected at Viral Diagnostic Laboratory (VDL), Shivammoga, Karnataka, India. These serum samples were collected in FTA Card, Fermentas, USA) and extracted viral RNA was brought to DRDE, Gwalior further stored at -80° C until use at High Containment Facility (BSL 3+) Defence Research & Development Establishment (DRDE) Gwalior. Along with this, apparently healthy serum samples were collected from healthy individuals at both sites VDL, Shivamogga and DRDE, Gwalior (n = 100) were kept as negative control.

The experimental protocols conducted in this study were approved by Defence Research and Development Establishment- Institutional Biosafety Committee, (DRDE-IBSC) vide no. IBSC/VIRO-02/20/PKD. The ethical approval & waiver from informed consent (For Infected and healthy individuals) was approved vide no. VCH/VEC/June-2021/04 of Vidya Ethics Committee, Gwalior, India.

### Viruses

In vitro transcribed KFDV RNA targeted against E gene was used as positive control in this study. In addition, the comparative analysis with other flaviviruses viz.; (West Nile virus (Eg 101), and dengue virus serotypes DENV-4, ND 73 (HM237348)), Japanese Encephalitis virus (JaOAr S982), Yellow fever vaccine strain (17D) and alphavirus virus; Chikungunya virus (EU 372006) was also used for cross-reactivity studies.

### RNA extraction

Viral RNA from clinical samples was extracted from 100 μl samples soaked on FTA card as per manufacturer’s instructions. Briefly the 2mm circular section was cut with the help of surgical blade and placed in elution buffer kept in 1.5 ml eppendorf tube. The tube was vortexed for 1 min and centrifuged further at 7871xg for 1 min. 140μl of clear supernatant was used as sample for viral RNA extraction employing QIAamp viral RNA mini kit (QIAGEN, Germany), according to the manufacture’s protocols. Briefly the 560μl of ethanol was added to the tube after 10 min of incubation in lysis buffer at room temperature. The whole mixture was passed through QIAspin columns at 7871xg for 1 min at 4°C. Further the column was washed with 500μl of wash buffer and viral RNA was eluted in 70 μl elution buffer. RNA was stored at -80°C till further use.

### In vitro transcription and quantitation of RNA

To get an amplified product with T7 promoter sequence was appended 5’ end of forward primer further this amplified product was used as template for in vitro transcription. IVT RNA was synthesized by exploiting T7 In vitro transcription kit (MBI Fermentas, USA). RNA pellet was resuspended in nuclease free water. Determination of copy number was carried out using the following formula-

$$Y\;molecules/\mu l=(Xg/\mu l\;RNA/[\;transcript\;length\;in\;nucleotides\;*\;340])\;*\;6.022\;*\;10^{23}$$

KFDV RNA was quantified using a nanodrop ND-1000 spectrophotometer (Thermo scientific, Germany). A later 10-fold serial dilution of the RNA transcript was used for determination of assay sensitivity. The construction of standard curve using Ct value obtained against the known concentration of serially diluted RNA.

### TaqMan real time RT-PCR

TaqMan real-time RT-PCR was standardized in DRDE, Gwalior using AgPath-ID^TM^ One-Step RT-PCR Reagents Kit (Thermos Fisher Scientific, USA) according to the reported oligos and probes as it is not commercially available. The assay was carried out in a final reaction volume of 25μl using 12.5μl of 2X RT-PCR buffer, 0.75μl of 25X RT-PCR enzyme mix, 0.5μl of each forward and reverse primers (50pm), 0.5μl of TaqMan probes and 5.25μl of nuclease free water. 5 μl of viral RNA was added as template. The thermal profile consists of 10 min of reverse transcription at 50⁰C one cycle and 2 min of polymerase activation at 95 ⁰C, followed by 40 cycles of PCR at 95 ⁰C for 15s and 60 ⁰C for 1 min. This assay was considered as a gold standard for detection of KFDV. All clinical samples (positive and negative panel used in this study) were tested with this assay.

### Probe free qRT-PCR

#### Primers designing

The oligonucleotide primers used for probe free RT-PCR amplification of KFDV were designed against envelope gene of KFD virus P9605 strain (GenBank number JF416958.1). The primer sets were selected and reanalyzed by aligning available KFDV full envelope gene sequence (n = 40) downloaded from NCBI GenBank database pertaining to different hosts, places, years. The sequences were aligned using ClustalW programme available in the Lasergene 5 package (DNAStar, USA). Conserved sequences across the KFDV envelope gene were located and cross checked during primer designing including variant strains 1957–2019.

#### Standardization of probe free qRT-PCR

Initial standardization was performed using the in vitro-transcribed KFDV E gene RNA (Fermentas, USA) further used as a positive control. For probe free qRT-PCR master mixture, 12.5μl of SYBR Green Power Up master mix (Applied Biosystems, USA), 0.5μl of primers-50pmol/μl (Eurofins, India), 0.5μl of RT/Taq enzyme and finally reaction volume was adjusted with 6μl nuclease free water. 5 μl of viral RNA was added as template. PCR without any template served as a negative control while invitro transcribed E gene RNA served as a positive control.

The thermal profile comprises of 30 min, reverse transcription at 50⁰C one cycle and 10 min of polymerase activation at 95⁰C, followed by 40 cycles of PCR at 95⁰C for 30 sec, 55⁰C for 1 min and 72⁰C for 30 sec. Following amplification, a melting curve analysis was performed to verify the authenticity of amplified product by its specific melting temperature (Tm) with the melting curve analysis software of the Applied Biosystem ABI 7,500 Dx (ABI, USA) according to manufacturer instructions. Briefly, the temperature was decreased to 57°C followed by incremental increase in temperature up to 95°C at a rate of 1°C/30 s/cycle with continuous measurement of fluorescence.

#### Dry- down probe free qRT-PCR

For dry-down formulation the concentration of cryoprotectant i.e. trehalose was optimized and was found 10% (w/v) trehalose to be the best. Preparation of 20μl dry-down formulation, 12.5μl of Single-Step 2X Power up master mix (Applied Biosystems, USA), 0.25μl of primers-50pmol/μl (Eurofins, India), 0.5μl of RT/Taq enzyme Platinum III-Invitrogen), 4μl of trehalose (10%, w/v) as lyoprotectants and 2.5μl nuclease free water was frozen at -80⁰ C for 2 h. following freezing, the tube were immediately placed under vacuum of 0.133 mBar pressures at -55°C for 3 h using a freeze dryer system (Labconco, USA). This formulation was prepared in 0.2ml polypropylene DNase/RNase free micro centrifugation tubes.

#### Stability studies of dry-down probe free qRT-PCR format

Dry-down formulation was stored at both room temperature and 4°C in desiccant containing sealed pouches. The stability of the reagents was studied at an interval of 1 week for 1 month and subsequently at 1 month interval up to 1 year.

The dry-down formulation was reconstituted with 20μl nuclease free water and mixed well using a pipette. After brief centrifugation at 1000x g for 1 min, it was subjected to qRT-PCR amplification by adding 5μl KFD RNA as template. PCR without any template severed as a negative control while in vitro-transcribed RNA served as a positive control.

#### Sensitivity of probe free qRT-PCR assay

The KFDV 216bp amplicon pertaining to the target within envelope gene (corresponding to genomic position 1,068–1,284) based primers were synthesized. The forward primer was labelled with T7 promoter. PCR product was used as template for IVT transcribed RNA. After in vitro transcription the RNA was purified finally by ethanol precipitation and was further resuspended in nuclease free water. The quantity of KFDV-RNA transcripts was determined using nanodrop and further the RNA molecular copies were calculated.

The detection limit of probe free qRT-PCR was determined using ten-fold serially diluted in vitro transcribed RNA, which was further added to probe free qRT-PCR assay along with positive and no template controls. The appearance of amplification curve along with melt curve was observed with real-time RT-PCR. Same master mixture was also carried out by conventional RT-PCR (C1000^TM^ Thermal Cycler PCR system (Bio-Rad, USA)) and visualized on 1% agarose gel analysis. The sensitivity of probe free qRT-PCR assay was then determined in both liquid format and dry-down (lyophilized) formats. Similarly the same dilution was also subjected to TaqMan qRT-PCR and conventional RT-PCR and the comparative results were observed.

#### Specificity of probe free qRT-PCR assay

The specificity of dry-down probe free qRT-PCR assay was determined by including other closely related flaviviruses (West Nile virus (Eg 101), and dengue virus serotypes DENV-4, ND 73 (HM237348)), Japanese Encephalitis virus (JaOAr S982), Yellow fever vaccine strain (17D) and alphavirus virus; Chikungunya virus (EU 372006). RNA extracted from flaviviruses and alphavirus infected culture supernatant (10^5−6^ RNA copies/rxn) were used as template.

#### Evaluation with clinical samples

The dry-down probe free qRT-PCR was evaluated using a panel of 50 KFDV positive samples and 100 apparently healthy samples. The assay was comparatively evaluated liquid format of probe free qRT-PCR assay and TaqMan qRT-PCR. The comparative evaluation results were compared manually by comparing Ct values observed in both the formats.

The clinical sensitivity and specificity of dry-down probe free qRT-PCR were determined and compared to TaqMan qRT-PCR and liquid probe free qRT-PCR.

### Statistical analysis

The standard deviation among samples was also calculated. Each experiment repeated three times. The analysis was performed using sigma stats one line tool employing one way anova. The values of significance was calculated for probe free dry-down qPCR in comparision to liquid format of TaqMan qPCR. The deviation between samples analyzed by three methods was calculated. Each experiment was repeated in a triplicate.

#### Nucleotide sequencing

Double pass sequencing of six KFD viruses from clinical samples were carried out with a Big dye terminator cycle sequencing ready reaction kit (Applied Biosystems, USA) on an ABI3130 sequencer following the manufacturer’s protocol. Following sequencing, the nucleotide sequences were edited and analyzed with the *EditSeq* and *MegAlign* modules of the Lasergene 5 software package (DNAStar Inc., USA).

## Results

### Primer designing

**Fig 1:**
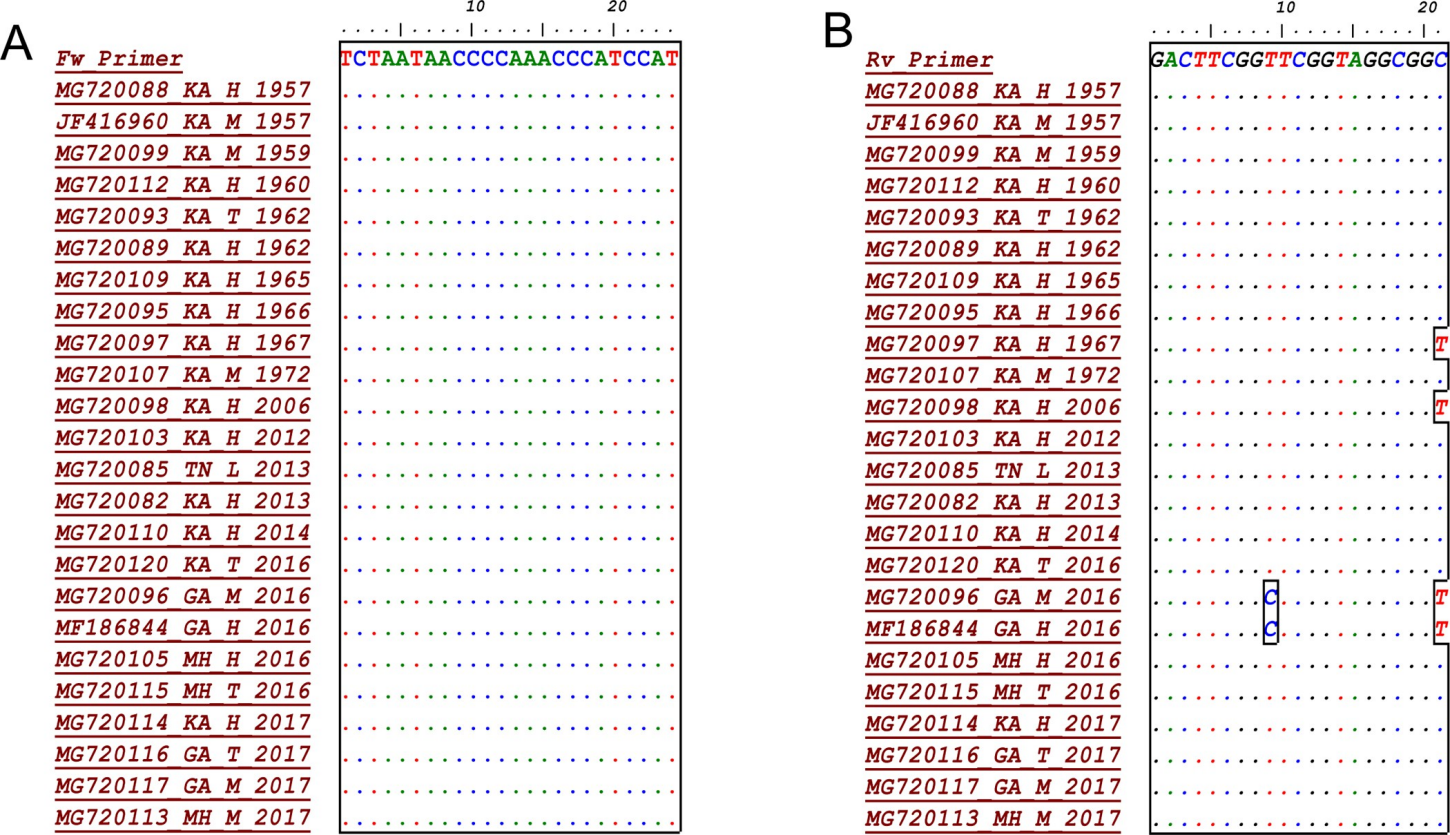
Multiple alignment of the E1-target regions (forward and reverse primers). Dot (·) indicates conserved nucleotides. GenBank accession numbers of representative sequences are specified on the left.

A highly specific primer sets were designed targeting Kyasanur Forest Disease Virus envelope gene. The available sequences were retrieved from GenBank includes sequences from inside and outside endemic zones comprises different hosts Ticks (T), Lengur (L), Human (H), Monkey (M) from 1957 to 2019 were analyzed after multiple sequence alignment for ascertaining conservancy depicted in Fig 1. The sequences of primers were detailed in Table 1.

**Table 1:** Details of primers for detection of Kyasanur Forest Disease Virus by probe free qRT-PCR.

Primer	Sequence (5’-3’)	Genomic Position
KFDV_FP	TCTAATAACCCCAAACCCATCCAT	1068–1091
KFDV_RP	GCCGCCTACCGAACCGAAGTC	1284–1264

### TaqMan real time RT-PCR

**Fig 2:**
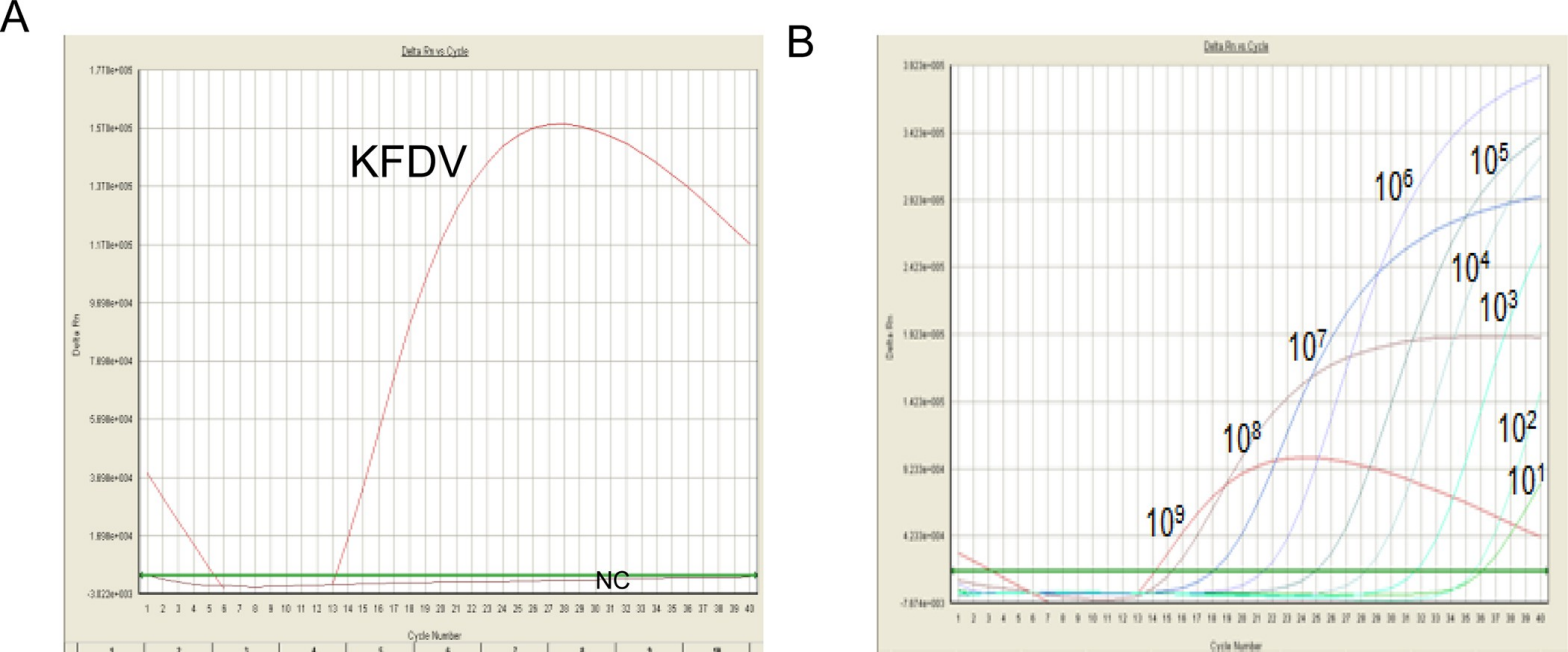
TaqMan qRT-PCR for KFDV. A. Showing KFDV specific amplification plot. B. Showing sensitivity of KFDV TaqMan qRT-PCR assay with 10 fold serially diluted IVT RNA; was found 10RNA copies/Rxn.

The detection limit of the KFD virus specific TaqMan qRT-PCR (reported prime probe) which was standardized in DRDE, Gwalior was found to be 10RNA copies/reaction (Fig 2). This assay was considered as a gold standard for the standardization and evaluation of in-house developed dry-down and liquid format probe free qRT-PCR assay.

### Probe free qRT-PCR in liquid format

**Fig 3:**
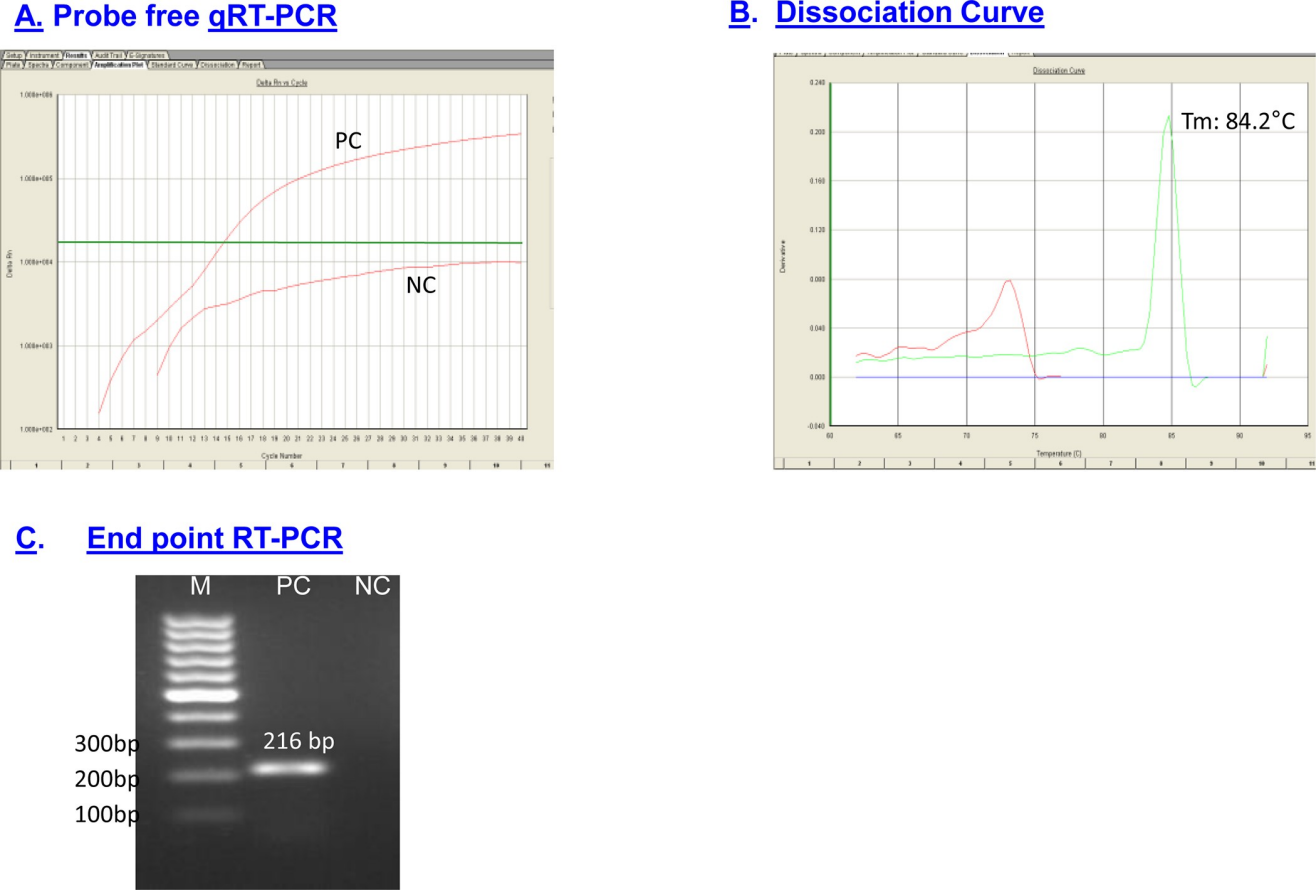
Probe free qRT-PCR (Liquid format) for KFDV. A. Showing KFDV specific amplification plot. B. Showing KFDV specific dissociation curve. C. Showing KFDV specific 216 bp Amplicon in conventional RT-PCR.

**Fig 4:**
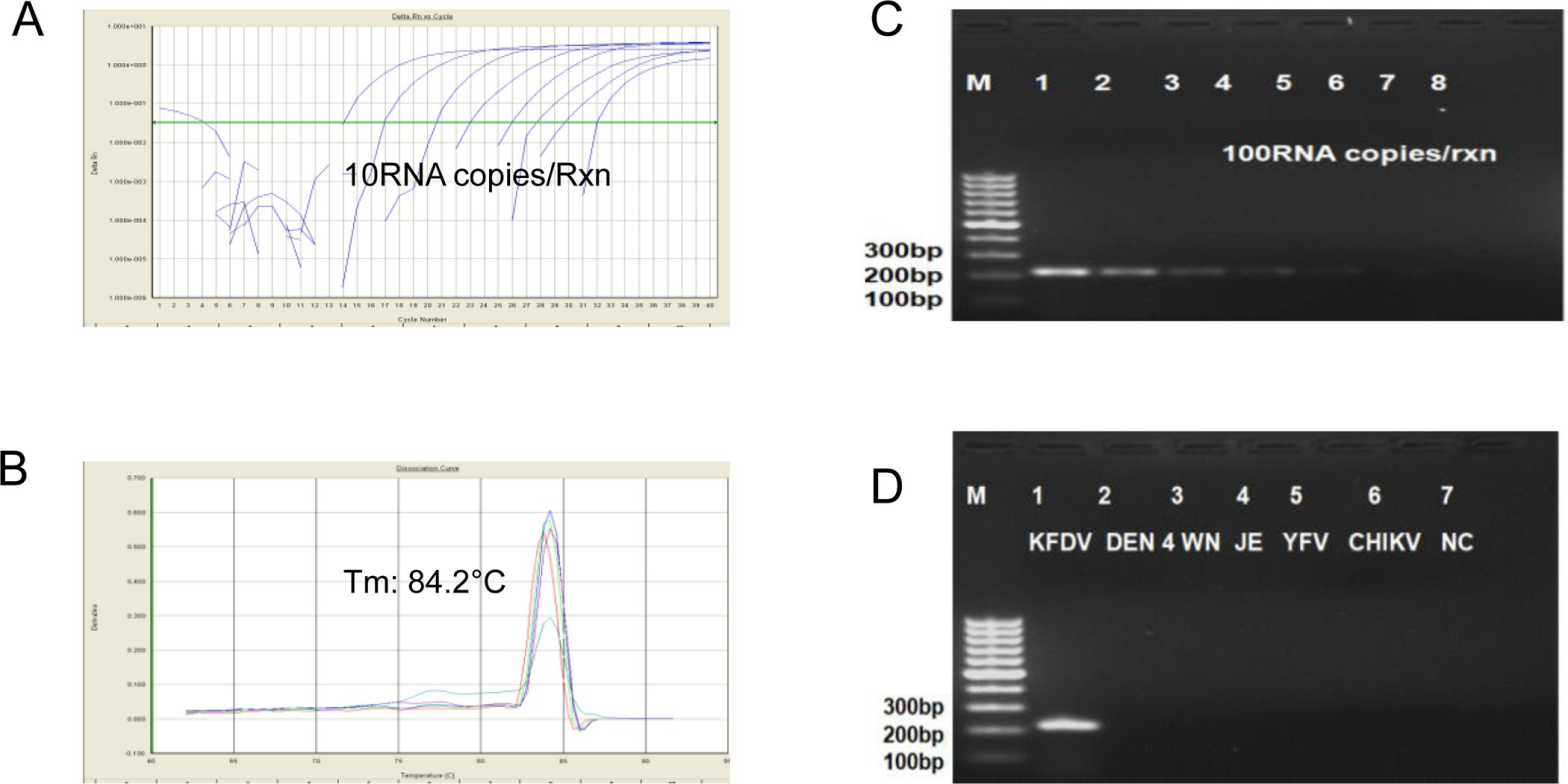
Sensitivity and specificity of dry down probe free qRT-PCR standard curve using IVT KFDV RNA with 300 ng/ul i.e. **1.6x 10**^**11**^ **RNA copies/μl.** A. Sensitivity was found to be 10RNA copies/Rxn for probe free qRT-PCR. B. Sensitivity 100RNA copies/Rxn for Conventional RT-PCR. C. KFDV specific dissociation curve. D. Specificity with closely related flaviviruses and alpha viruses.

A probe free qRT-PCR targeting KFD virus envelope gene was best optimized at Tm55°C and specific dissociation was obtained at 84°C. The assay was first standardized in liquid format for both qRT-PCR as well as conventional RT-PCR (Fig 3A–3C). The detection limit of the probe free qRT-PCR assay was found to be 10RNA copies/reaction, which was same as TaqMan qRT-PCR (Fig 4A). At the same time the detection limit of conventional RT-PCR was found to be 100RNA copies/reaction (as it is 1000 times less sensitive than real time RT-PCR) (Fig 4B). The standard curve for KFDV constructed using Ct values obtained against the known concentration of 10-fold serial dilutions of KFDV in vitro transcribed RNA exhibits a linear curve with the coefficient of correlation R^2^ = 0.997. Melting curve analysis with the melting curve analysis software of the ABI 7,500Dx (ABI, USA) showed that specific amplicon melts at 84.4°C (84.1–84.6°C) KFDV-specific amplification and dissociation are depicted in Fig 4C.

It was also observed that the KFDV-specific primer pair was highly specific in the detection of KFDV having no cross reactivity with any of the above mentioned flaviviruses and alphavirus used in this study, thereby establishing the specificity of the primer set for KFDV Fig 4D. Besides, no cross reactivity was also observed with the viruses of flaviviruses and alphavirus group as mentioned above, associated with viral hemorrhagic fever having similar clinical picture.

### Dry-down probe free qRT-PCR

**Fig 5:**
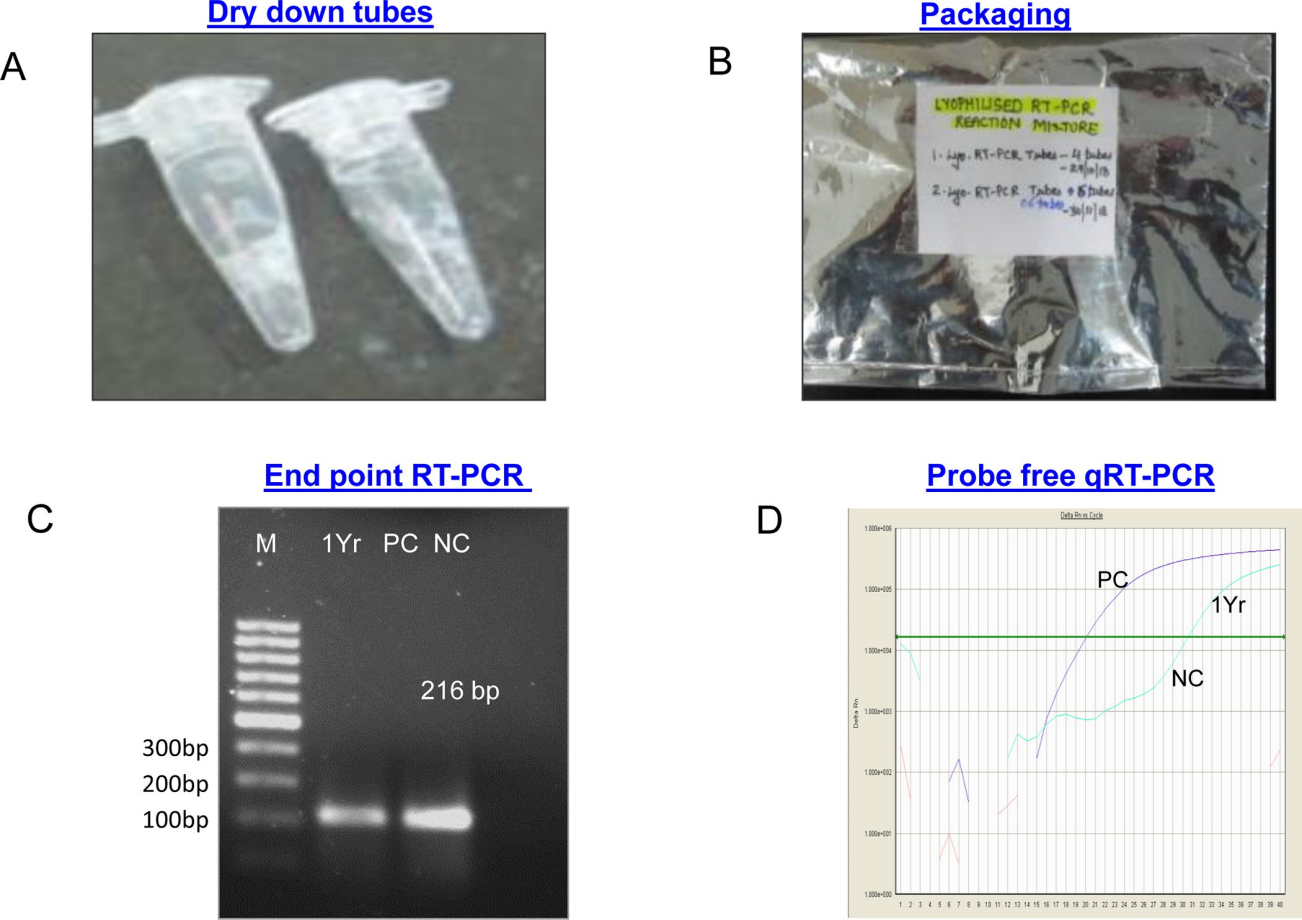
Storage and stability studies of dry-down probe free qRT-PCR. A). Post dry–down PCR tubes containing lyophilized probe free qRT-PCR master mix. B). Packaging of dry-down formulated tube along with desiccators in a sealed aluminium pouch. C). Stability of dry down probe free qRT-PCR in *end point* PCR after 1 Year of storage. D).Stability of dry down probe free qRT-PCR in qRT-PCR after 1 Year of storage.

Further the similar liquid format of probe free qRTPCR was standardized for thermal stability by adding 10% trehalose as a cryoprotectant (optimized in our lab). Later it was subjected to lyophilisation for 3–4 hours at vacuum of 0.133 mBar pressure using a freeze dryer system (Labconco, USA). The reaction mix were lyophilized in batches and further studied for thermal stability at room temperature and 4°C (Fig 5A and 5B). The detection limit of the dry-down probe free qRT-PCR format was found to be 10RNA copies/reaction similar to that of liquid probe free qRT-PCR format. At the same time the detection limit of dry-down conventional RT-PCR was found to be 100RNA copies/reaction which was similar to liquid conventional RT-PCR format. The stability was found 7 days at room temperature and up to 1 year achieved at 4° C in both dry-down conventional and qRT-PCR formats without any significant changes with compare to liquid formats of KFDV detection assays (Fig 5C and 5D).

No difference in the analytical sensitivity were found between the results obtained with the gold standard and those obtained by the dry-down format, with 100% detection rates obtained with both the methods. The results obtained in the C_t_ values recorded by the liquid format and the dry-down format exhibit a 0.5 C_t_ difference (Table 2). The diagnosis specificity of the two formats (liquid and dry-down) was 100%.

**Table 2:** Comparative evaluation of liquid and dry-down probe free qRT-PCR with TaqMan qRT-PCR in clinical samples.

Dry-down Probe free qRT-PCR	TaqMan qRT-PCR		
	Positive	Negative	Total
**Positive**	50	0	50
**Negative**	50	50	50
**Total**	100	50	100

Concordance = 100%, Sensitivity = 100%, Specificity = 100%

### Comparative evaluation of liquid and dry-down probe free qRT-PCR with samples

**Fig 6:**
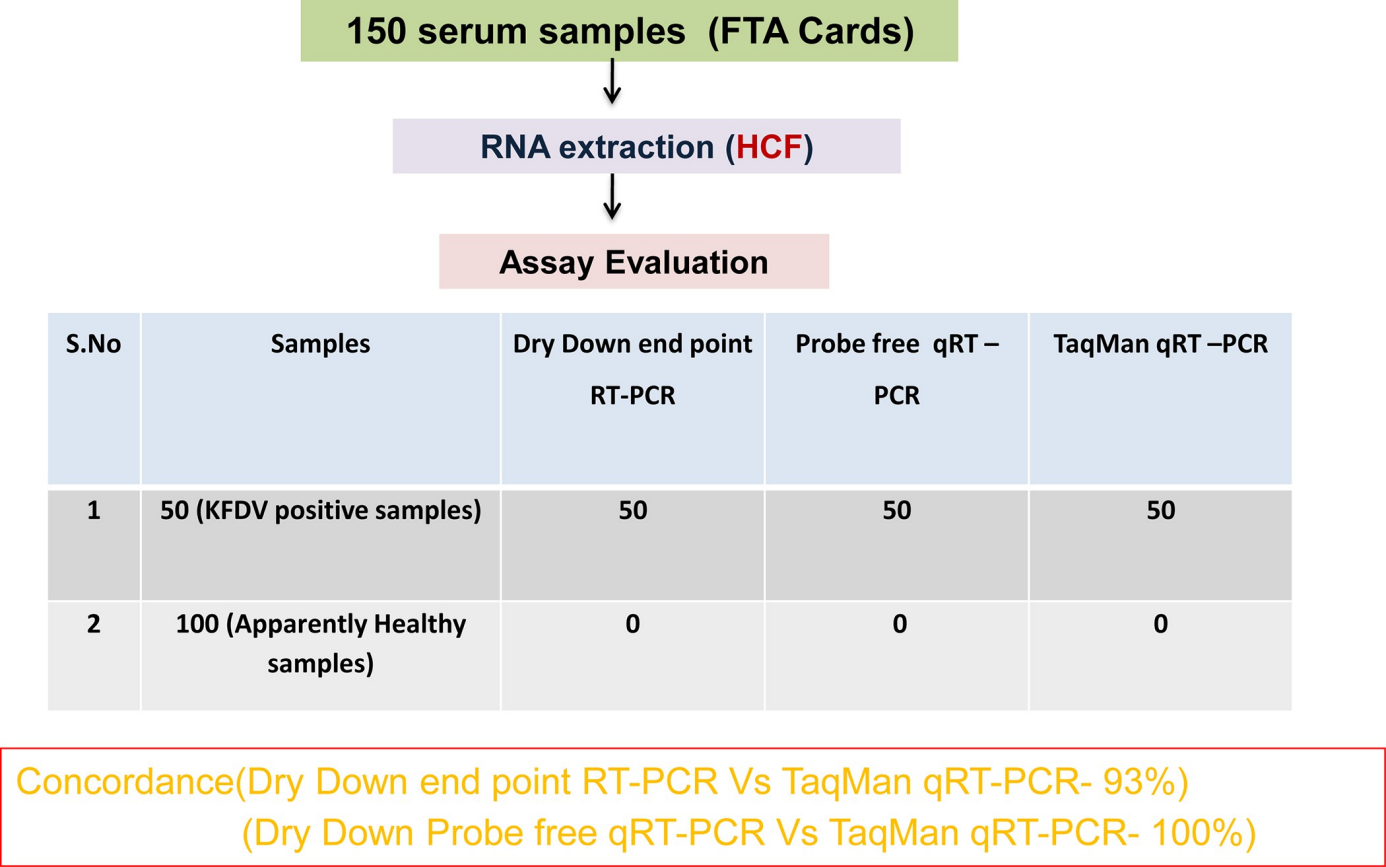
Comparative evaluation with clinical samples. Showing comparative evaluation of dry down probe free RT-PCR in both conventional and qRT-PCR formats compared with TaqMan qRT-PCR.

A panel of total 150 samples including 50 KFDV sample (Human, Ticks, Monkey) and 100 apparently healthy serum samples was used for the clinical evaluation studies of dry-down probe free qRT-PCR. On comparison of the results of liquid and dry-down probe free qRT-PCR with TaqMan qRT-PCR revealed 100% concordance. The results obtained with the evaluation with sample in the C_t_ values recorded by the liquid format and the dry-down format (Table 3). The diagnosis specificity of the two formats (liquid and dry-down) was 100%. Out of 50 positive samples all 50 were found positive by both the methods while 5 samples of low viral load (Ct value above 35) were missed by the conventional RT-PCR indicating the sensitivity of 93% (as it is 1000 times less sensitive than qRT-PCR). However, none of the negative samples showed any signal of positivity, indicating a sensitivity of 100% (Fig 6).

**Table 3:** Comparative evaluation of liquid and dry-down probe free qRT-PCR, conventional RT-PCR Vs TaqMan qRT-PCR in KFDV clinical samples.

S.No.	Sample ID	TaqMan qRT-PCR	Probe free qRT-PCR (liquid format)	Probe free qRT-PCR (dry-down format)	Conventional RT-PCR
1	K-1	34	34	33.5	-
2	K-2	27	27.1	26.6	+
3	K-3	30.51	30.51	30.23	+
4	K-4	34.07	35.07	35.67	-
5	K-5	30.61	30.61	31.12	+
6	K-6	24.8	24.8	24.87	+
7	K-7	30.49	30.49	29.93	+
8	K-8	35.32	35.32	35.91	-
9	K-9	24.9	24.9	24.2	+
10	K-10	26.5	26.5	25.9	+
11	K-11	20	20	21.1	+
12	K-12	34.9	34.9	34.5	+
13	K-13	30.5	30.5	30	+
14	K-14	36	36	36.2	-
15	K-15	33.2	33.2	33.6	+
16	K-16	32	32	33	+
17	K-17	28	28	28.9	+
18	K-18	29	29	29.6	+
19	K-19	21	21	22.1	+
20	K-20	27	27	26.9	+
21	K-21	23	23	23.6	+
22	K-22	29.5	29.5	29	+
23	K-23	35.6	35.6	36.2	-
24	K-24	27	27	27.4	+
25	K-25	26	26	26.4	+
26	K-26	29	29.2	29.1	+
27	K-27	25	26	25.7	+
28	K-28	22	22.3	22	+
29	K-29	19	19.1	19.1	+
30	K-30	20	20	20	+
31	K-31	29	29.2	29.1	+
32	K-32	31.2	29	29.8	+
33	K-33	28	28.06	28.76	+
34	K-34	31.5	31	31.2	+
35	K-35	30	30.27	30.16	+
36	K-36	31	32.1	32.2	+
37	K-37	34	35	35.1	+
38	K-38	27	27.59	27.4	+
39	K-39	28	28.17	28	+
40	K-40	32	32.5	32.7	+
41	K-41	26	26.62	26.52	+
42	K-42	29	30.29	30.1	+
43	K-43	26	26.7	26.2	+
44	K-44	23	23.69	23.8	+
45	K-45	28	28.7	28.9	+
46	K-46	33	34	33.9	+
47	K-47	31	31.3	31.5	+
48	K-48	29	29.6	29.6	+
49	K-49	26	26.5	26.4	+
50	K-50	27.9	27.8	27.5	+

NOTE: 26 and 27- tick samples

28 and 29- monkey sample

30- cell culture fluid

## Statistical analysis

**Fig 7:**
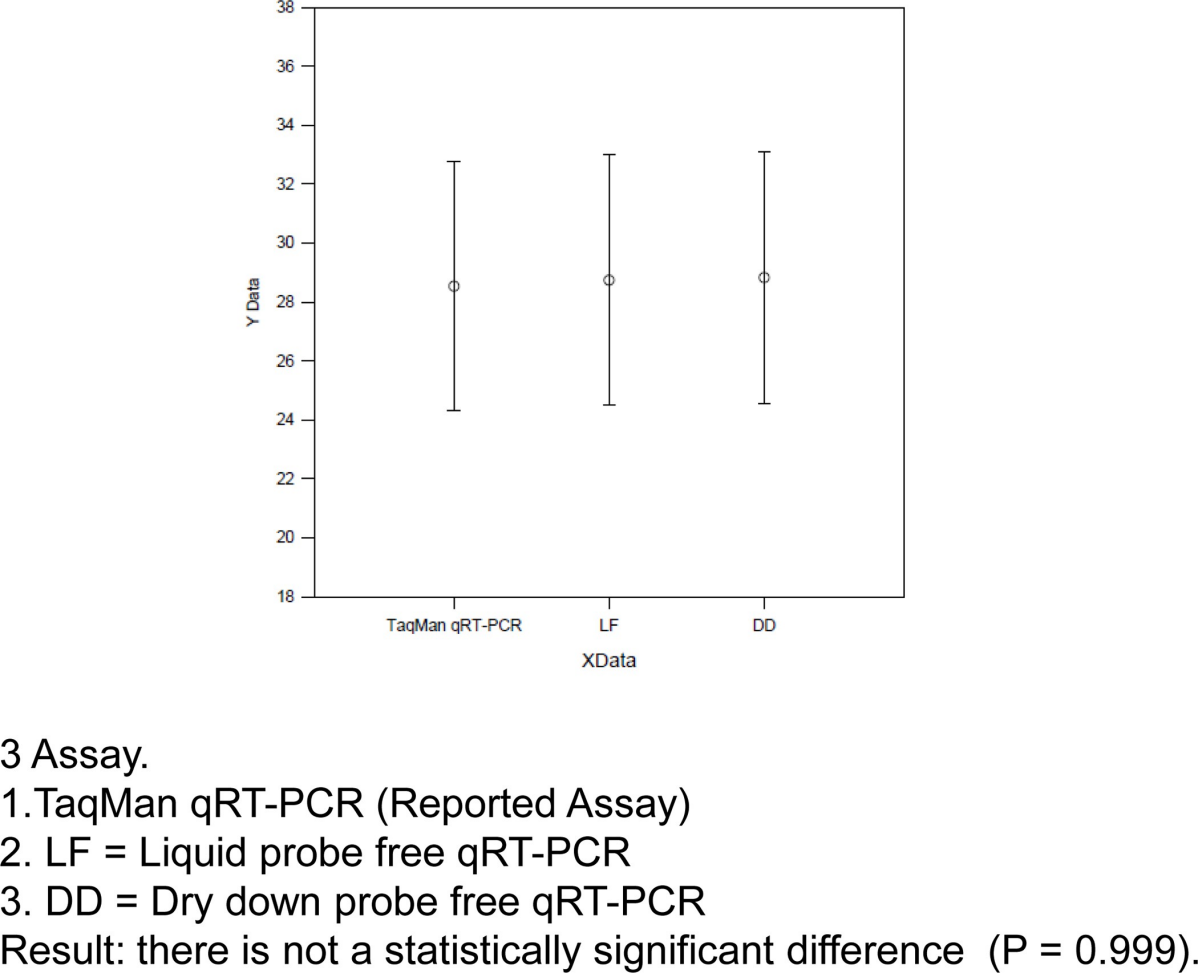
Statistical analysis of comparative evaluation of SYBR probe free dry down q-PCR Vs TaqMan q-PCR.

The statistical analysis revealed there is no such statistical difference between the TaqMan qRT-PCR, liquid probe free qRT-PCR and dry-down probe free qRT-PCR (p value = 0.984) using Sigma stat, one way annova (Fig 7). The precision value was found 1.0 for both the assay in compare to TaqMan qPCR.

### Nucleotide sequencing

Six samples which were found positive for probe free qRT-PCR and conventional RT-PCR were subjected to sequencing by Sanger’s sequencing method. BLAST analysis revealed 100% nucleotide identity with 99% sequence coverage to Kyasanur Forest Disease Virus envelope genomic region in the specific products. Sequencing results thus confirmed the accuracy of probe free qRT-PCR in detecting KFDV in samples.

## Discussion

Kyasanur Forest Disease is a highly pathogenic member of the family *Flaviviriadae* causing morbidity and mortality in humans and monkey populations [7, 8]. KFDV is widely distributed in southern regions of India and is progressing toward neighbouring districts of the Western Ghats covering states of Gujarat, Maharashtra, Goa, Karnataka, Tamil Nadu and Kerala [9]. The spread of KFD to newer areas is a major cause of concern which is probably due to the migration of monkey and small rodents, which harbor the virus [10]. So, large scale diagnostic study should be conducted for the identification of the virus.

The present study described the strategic development of a freeze-dried, ready-to-use probe free qRT-PCR master mix based on specific detection of the envelope structural gene which is highly conserved in KFDV. This dry- down formulations provides stability to reagents against temperature fluctuation and storage conditions which is very critical for storage of qPCR reagents in molecular diagnosis. We have first standardized and evaluated a probe free qRT-PCR assay for rapid detection of KFDV virus E gene target. After achieving sensitivity limit which was found comparable to a reported TaqMan qRT-PCR, we have optimized a dry-down conditions for theses reagents with the add of stabilizers. The reagents stability post dry-down was found comparable to liquid format. The assay was evaluated with a panel of KFDV positive and apparently healthy sample panel revealed good correlation between dry-down probe free qRT-PCR and TaqMan qRT-PCR. However five samples belong to high Ct values were found to be missed in conventional PCR due to lower sensitivity of assay in comparison to real time PCR.

This probe free qRT-PCR master mix was prepared in 0.5ml PCR tubes and was freeze at -80° C. Further lyophilisation under vacuum with the aid of cryoprotectant was studied for reagents stability to temperature fluctuations. Add of sugar additives were well documented in molecular research challenging to effectual freeze- drying of the master mix without any loss in functionality of the PCR reagents due to its low hygroscopity [11, 12]^.^

In addition to this, the challenges faced in transportation and storage of PCR diagnostic kits one step ready-to-use dry-down probe free qRT-PCR formulation is a bit reliable as it reduces the time consumed for reaction preparation and also reduces the chances of contamination. The shelf-life was found to be 7 days at room temperature (28⁰C-30⁰C) while long term stability of up to 1 year was achieved at 4° C without any loss of amplification performance of the desired region of KFDV.

The results supported the real-time utility of the dry-down probe free formulation as described in this study as highly reliable diagnostic tool in point-of-care laboratories as well as in-field investigation of KFDV, wherein the specificity and sensitivity of the assay will not be compromised. The KFD is an important and highly prevalent infection restricted to southern part of India. The occurrence of outbreaks occurs in these limited areas. Therefore, very limited samples could be shared and made available for evaluation of diagnostic assay. KFD is high-risk pathogens and handling of ticks and monkeys’ samples would require Biocontainment facility. During COVID-19 pandemic a huge number of laboratories are equipped with RT-PCR testing system in India which may fulfill need of biocontainment facility."

Comparatively various diagnostics assays are available, but this is cost effective diagnostic assay which reduces the strict cold chain maintenance during storage and transportation. Also the aid of dry-down probe free qRT-PCR in both conventional as well qRT-PCR formats has advantage of user choice applicability over the probes based costly TaqMan qRT-PCR. In addition to this it avoids manual error of repeated pipetting in liquid phase PCR, freeze thawing of highly sensitive PCR reagents thus giving promising results in field applications. This is low cost as compared to TaqMan qPCR and serve as an alternate to the reagent lability issues under field conditions. The method is sensitive, specific and enables to detect KFD virus. The assay found highly concordant with TaqMan qPCR. Thus this dry-down probe free PCR will serve as an alternated detection platform in rapid diagnosis of KFD.

This dry format construction has great potential for a diagnostic program in a variety of industries such as the medical, veterinary and agricultural industries. Keeping in mind the recent advances in molecular diagnostics it is assumed that the dry-down format is widely recognized due to its fast, non-hassel, low chance of contamination, critical detection limit that can improve all diagnostic time and quality.

## Supporting information

S1 Fig KFDV probe free qRT-PCR primers multiple sequence alignment. (PDF)

S2 Fig KFDV TaqMan qRT-PCR. (PDF)

S3 Fig Probe free qRT-PCR assay. (PDF)

S4 Fig Sensitivity and specificity of dry down probe free qRT-PCR. (PDF)

S5 Fig Storage and stability studies of dry down probe free qRT-PCR. (PDF)

S6 Fig Comparative evaluation with clinical samples. (PDF)

S7 Fig A) Statistical analysis of the developed Assays (liquid and dry down format) with reported assay (TaqMan qRT-PCR); B) Analysis Summary.
(PDF)
