# Supplementary figures and images for: Dry- down probe free qPCR for detection of KFD in resource limited settings
Source: PLoS One. 2023 May 10;18(5):e0284559. doi: 10.1371/journal.pone.0284559 (PMC10171661; doi:10.1371/journal.pone.0284559)

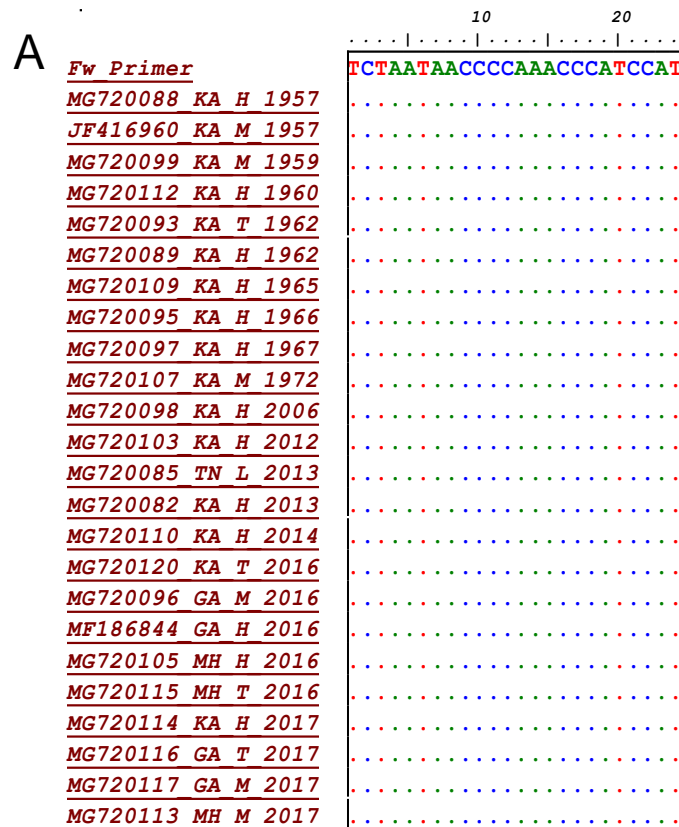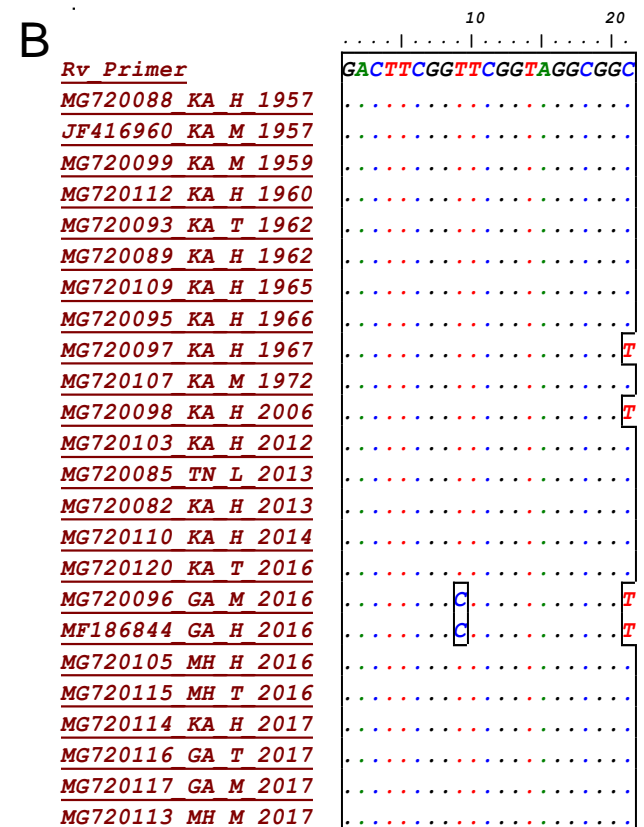

**S1 Fig KFDV probe free qRT- PCR primers Multiple Sequence Alignment**

Supplement: S1 Fig — (PDF) [file pone.0284559.s001.pdf]

### A. Probe free qRT-PCR

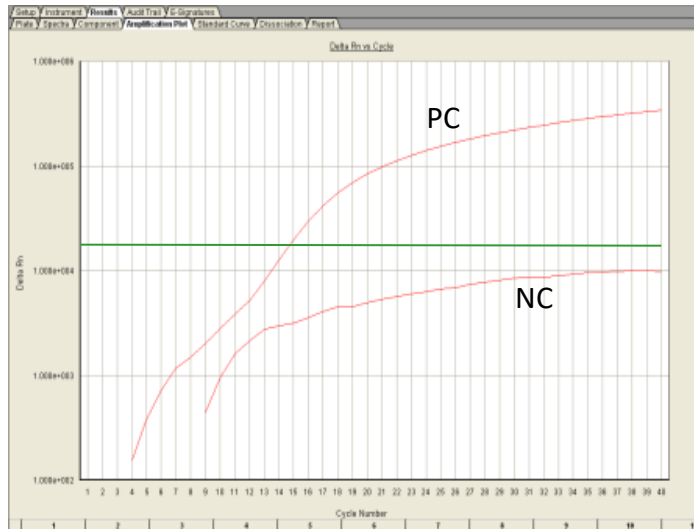

### B. Dissociation Curve

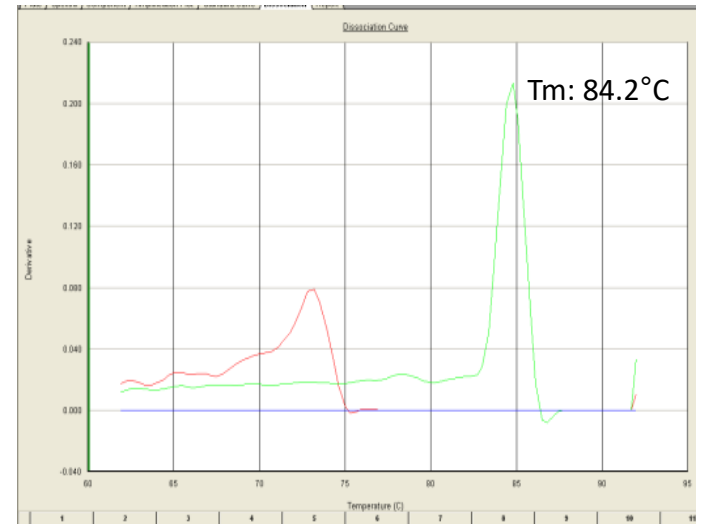

### C. End point RT-PCR

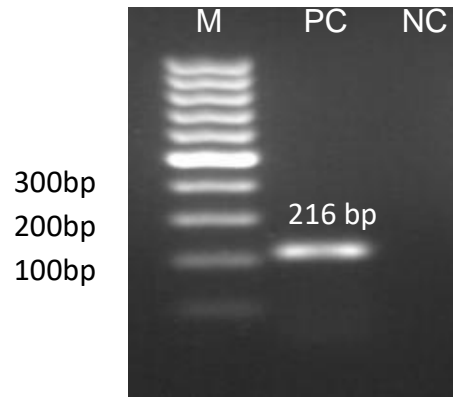

**S3 Fig . Probe free qRT-PCR assay**

Supplement: S3 Fig — (PDF) [file pone.0284559.s003.pdf]

A

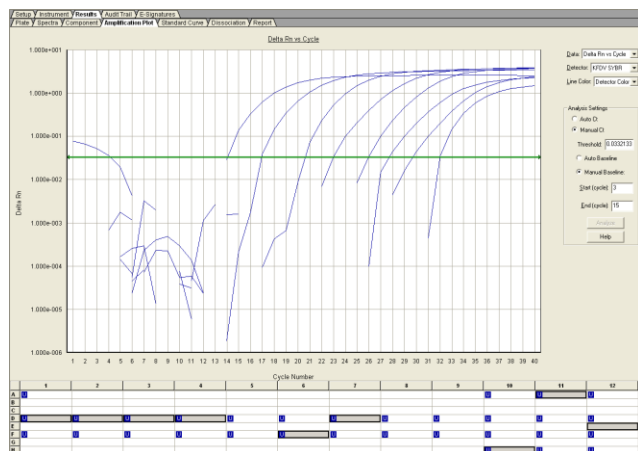

C

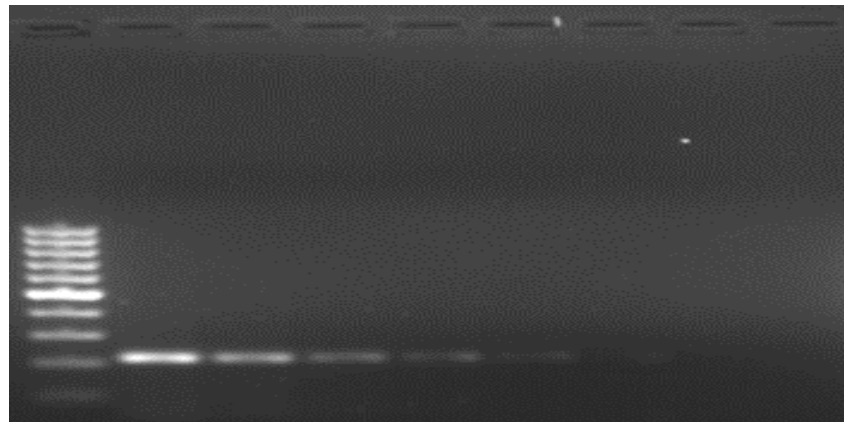

B

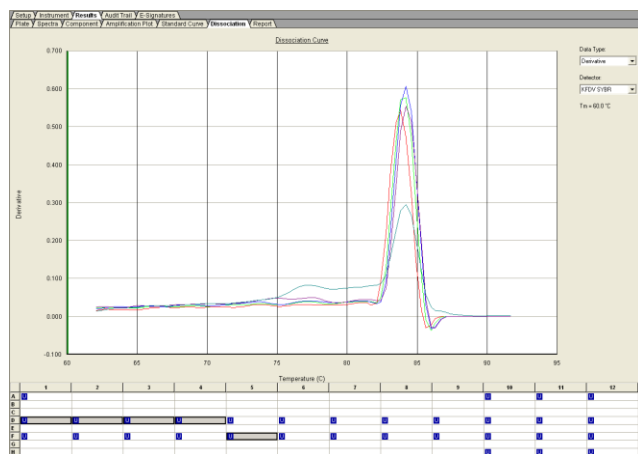

D

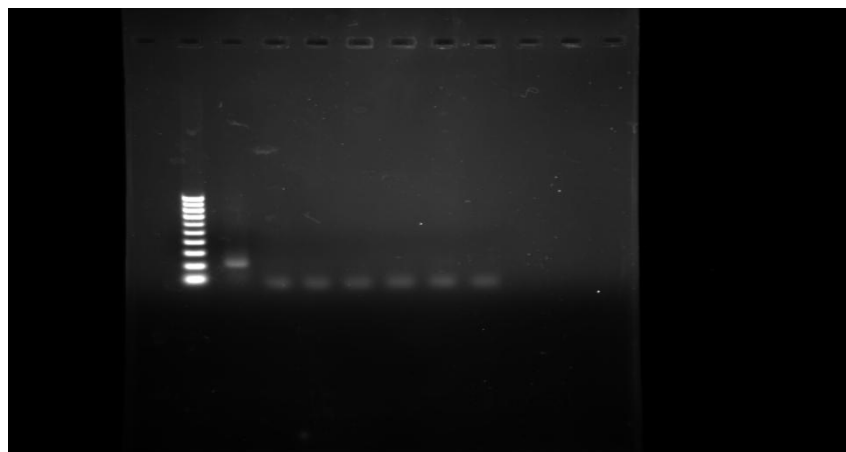

**S4 Fig : Sensitivity and specificity of dry down probe free qRT-PCR**

Supplement: S4 Fig — (PDF) [file pone.0284559.s004.pdf]
