# Supplementary material for: Dry- down probe free qPCR for detection of KFD in resource limited settings
Source: PLoS One. 2023 May 10;18(5):e0284559. doi: 10.1371/journal.pone.0284559 (PMC10171661; doi:10.1371/journal.pone.0284559)
Supplement: S2 Fig — (PDF) [file pone.0284559.s002.pdf]

A

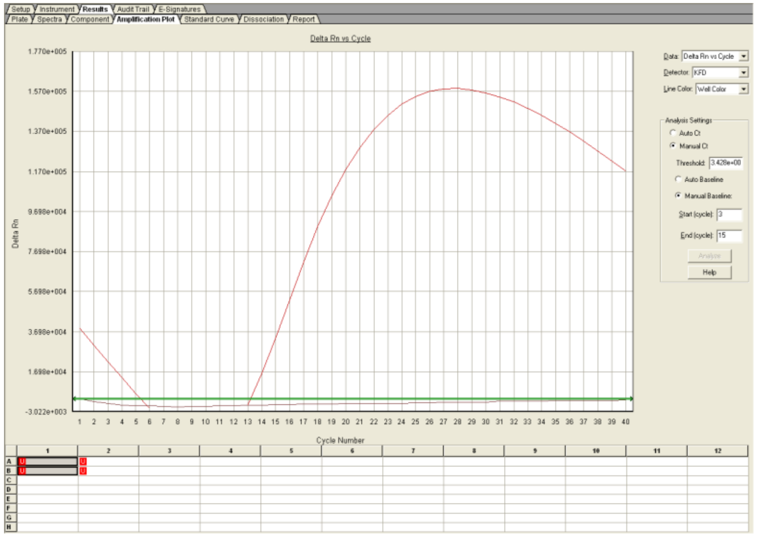

KFDV dilution 8 june 2018.sds
Amplification Plot

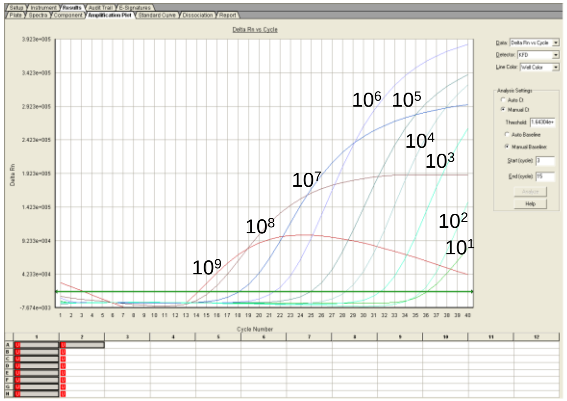

Qubit Fluoremeter: 470 ng/ µl; 1.28 X 10 ^{11} Copies/µl plasmid

S2 Fig KFDV TaqMan qRT- PCR
