# Supplementary material for: Dry- down probe free qPCR for detection of KFD in resource limited settings
Source: PLoS One. 2023 May 10;18(5):e0284559. doi: 10.1371/journal.pone.0284559 (PMC10171661; doi:10.1371/journal.pone.0284559)
Supplement: S5 Fig — (PDF) [file pone.0284559.s005.pdf]

Dry down tubes

Packaging

RAW

A

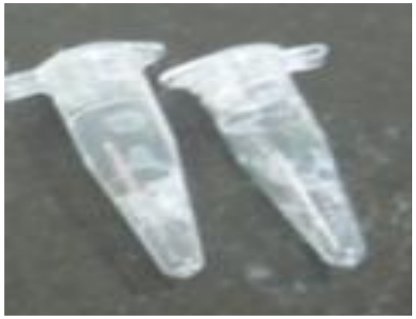

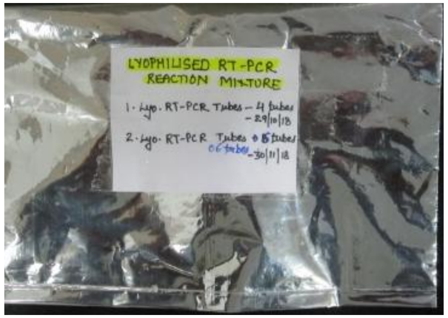

End point RT-PCR

Probe free qRT-PCR

C

End point RT-PCR

Probe free qRT-PCR

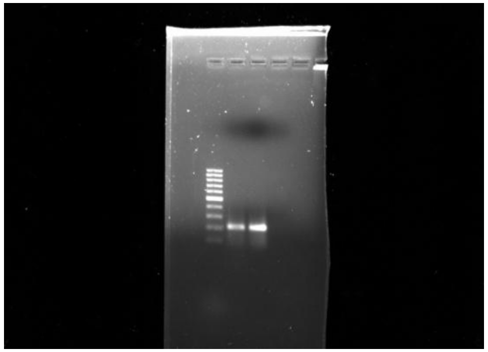

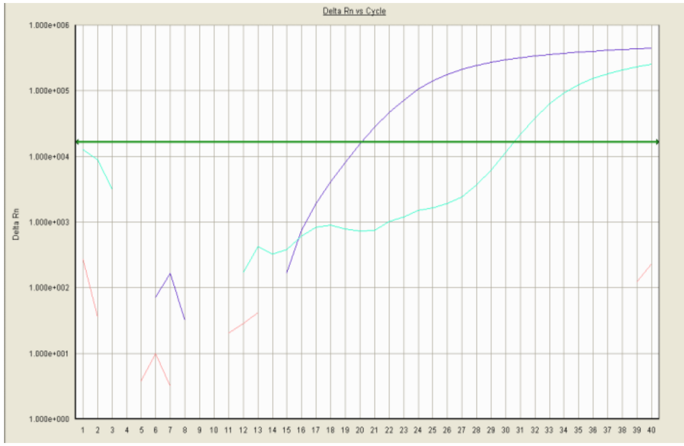

S5 Fig : Storage and stability studies of dry down probe free qRT-PCR
