# Supplementary material for: Dry- down probe free qPCR for detection of KFD in resource limited settings
Source: PLoS One. 2023 May 10;18(5):e0284559. doi: 10.1371/journal.pone.0284559 (PMC10171661; doi:10.1371/journal.pone.0284559)
Supplement: S6 Fig — (PDF) [file pone.0284559.s006.pdf]

150 serum samples (FTA Cards)

↓

RNA extraction (HCF)

↓

Assay Evaluation

S.No	Samples	Dry Down end point RT-PCR	Probe free qRT –PCR	TaqMan qRT –PCR
1	50 (KFDV positive samples)	50	50	50
2	100 (Apparently Healthy samples)	0	0	0

Concordance(Dry Down end point RT-PCR Vs TaqMan qRT-PCR- 93%)
(Dry Down Probe free qRT-PCR Vs TaqMan qRT-PCR- 100%)

S6 Fig : Comparative evaluation with clinical samples
