# Supplementary material for: Dry- down probe free qPCR for detection of KFD in resource limited settings
Source: PLoS One. 2023 May 10;18(5):e0284559. doi: 10.1371/journal.pone.0284559 (PMC10171661; doi:10.1371/journal.pone.0284559)
Supplement: S7 Fig — A) Statistical analysis of the developed Assays (liquid and dry down format) with reported assay (TaqMan qRT-PCR); B) Analysis Summary. (PDF) [file pone.0284559.s007.pdf]

RAW

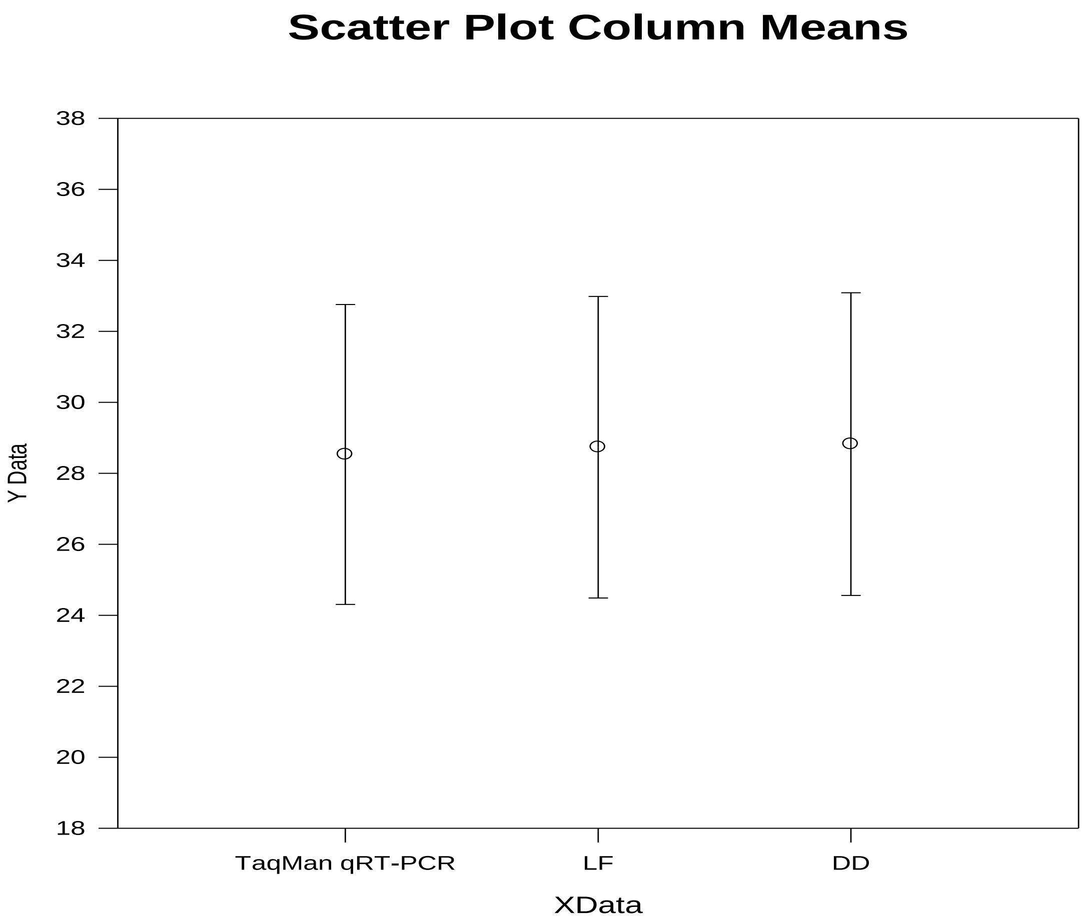

S7 Fig: Statistical analysis of the developed Assays(liquid and dry down format) with reported assay (TaqMan qRT-PCR)

S7 Fig:B) Fig Analysis Summary

One Way Analysis of Variance **Wednesday, December 21, 2022, 10:47:04**

Data source: Data 1 in Notebook 1.SNB

Normality Test: **Passed** **(P = 0.207)**

Equal Variance Test: **Passed** **(P = 0.999)**

**Group Name**		**N**	**Missing**	**Mean**	**Std Dev**	**SEM**
Taq Man qRT-PCR		50	0	28.530	4.224	0.597
LF	50	0	28.734	4.248	0.601	
DD	50	0	28.823	4.264	0.603	

**Source of Variation**		**DF**	**SS**	**MS**	**F**	**P**
Between Groups		2	2.261	1.130	0.0627	0.939
Residual	147	2649.640	18.025			
Total	149	2651.900				

The differences in the mean values among the treatment groups are not great enough to exclude the possibility that the difference is due to random sampling variability; there is not a statistically significant difference (P = 0.939).

Power of performed test with alpha = 0.050: 0.049

The power of the performed test (0.049) is below the desired power of 0.800.
Less than desired power indicates you are less likely to detect a difference when one actually exists.
Negative results should be interpreted cautiously.
